# Synthesis and characterization of manganese ferrite from low grade manganese ore through solid state reaction route

**DOI:** 10.1038/s41598-021-95625-z

**Published:** 2021-08-10

**Authors:** Salar Ahmad, Sajjad Ali, Ikram Ullah, M. S. Zobaer, Ashwag Albakri, Taseer Muhammad

**Affiliations:** 1 Materials Research Laboratory, Department of Physics, University of Peshawar, Peshawar, 25120 KP Pakistan; 2 Department of Sciences and Humanities, National University of Computer and Emerging Sciences Peshawar Campus, Peshawar, 25000 KP Pakistan; 3 McGovern Medical School, The University of Texas Health Science Center at Houston, Texas, TX USA; 4 Department of Computer Science, College of Computer Science & Information Technology, Jazan University, Jazan, 45142 Saudi Arabia; 5 Department of Mathematics, College of Sciences, King Khalid University, Abha, 61413 Saudi Arabia

**Keywords:** Structural materials, Chemical physics

## Abstract

Manganese ferrite spinel has been synthesized by using low grade manganese ore and ferric oxide as sources of manganese oxide and iron oxide through solid state reaction route by taking manganese and iron mole ratio of 1:2 respectively. The impact of sintering temperature on phase composition and particle size is investigated. Similarly, the impact of frequency on dielectric constant, dielectric loss, AC (alternating current) conductivity and tangent losses is also investigated. The results shows the presence of spinel structure manganese ferrite (MnFe_2_O_4_) as the major phase for the sample sintered at 1200 °C. It has been established that the crystallite size increase with rise in sintering temperature. The surface morphology of the sample sintered at 1200 °C show pyramidal and triangular shape grains. The dielectric constant (εʹ) and dielectric losses (εʹʹ) were observed to decrease with increasing the sintering temperature and frequency. Furthermore, the AC (alternating current) conductivity was found to rise with rise in applied frequency. On the other hand, the tangent losses falls considerably with rise in applied frequency.

## Introduction

Spinel ferrites (MFe_2_O_4_), where M^2+^ is divalent metal cation, are a significant class of magnetic materials, which have been explored the most in recent couple of decades because of good combination of electrical and magnetic properties. The materials draw in the analyst enthusiasm because of innovative variables of large electrical resistivity, small eddy current losses, large value of dielectric constants, high initial permeability and moderate saturation magnetization^1^. Polycrystalline ferrites are generally utilized in electronic uses in a scope of frequencies reaching out from microwave to radio frequency^2^. They are significant financially in light of the fact that they can be applied in numerous gadgets, for example, stage shifter, large frequency transformer cores, switches, resonators, PCs, TV and cell phones^3,4^. Ferrites mostly form the cubic spinel type structure, cation between two different interstitial lattice sites, tetrahedral (A) and octahedral (B) sublattices^2^. The divalent metal cation M^2+^ can occupy either (A) or (B) site, or both of the spinel structure. It can be commonly represented as AB_2_O_4_. Among the spinel ferrites, the MnFe_2_O_4_ is a soft magnetic semi conducting material characterized by low dielectric losses and high resistivity because of its dielectric nature^2^. It is verifiable truth that the dielectric properties of ferrites are emphatically needy upon temperature and frequency. Ashraf et al.^5^ studied the effect of adding magnetic nano-particles into biofluidic medium like blood and successfully modeled the flow model at low Reynolds number. As an application, the synthesized Mn-ferrite nano-particles could also be used for the same purpose.

Synthesis of Ferrite nano particles is of great interest because of its antiproliferative activity which is affected by specific surface area and average crystal size. Almessiere et al.^6^ obtained ferrite nanoparticles by solgel and ultrasonic methods and found that the samples obtained via ultrasonic methods are more effective against cancer cells. Kozlovskiy et al.^7^ contemplated the impact of annealing temperature on synthesis of ferrite nanowires and found that the oxygen dissemination within the structure of nanowires is not uniform along with formation of oxide compounds for annealing temperature range of 400 to 600 °C and the corresponding distribution is uniform with formation of oxide phases for annealing temperature of 800 °C. wakif et al.^8^ studied the magneto-convection process under heat influence and found that the thermo-magneto-hydrodynamic feature depends on electric properties and size of the nanoparticles shows a destabilizing effect. Trukhanov et al.^9^ obtained hexaferrite nano particles by sol gel method with sintering temperature of 600–1100 °C and found that the average particle size and specific surface area of the obtained samples increases with increase in temperature. Singh et al.^10^ prepared hexaferrite nano particles by a two route ceramic technique and found that doping of Cr^+3^ and Co^+2^ reduce thickness and increase the absorption which is attributed to input impedance and eddy current losses. Zdorovets et al.^11^ obtained oxide nanostructures by annealing temperature methods and found an increase in the resource lifetime for the material sintered at 500 °C. Thumma et al.^12^ studied the migration of nano-particles dur to temperature gradient i.e. heat sintering and found that certain parameters tends to decrease the temperature and hence the concentration profile. Wakif et al.^13^ found that the heat transfer rate decreases as the strength of the magnetization field increases. Qasim et al.^14^ investigated that at large values of thermal conductivity, the temperature profiles decreases. Pande et al.^15^ synthesized Mn-ferrite nanoparticles via coprecipitation method and found that the obtained nanoparticles have single crystalline phase with best magnetic properties. Wakif et al.^16^ found that with the increase in nanoparticles concentration, thermal resistance increases. Qasim et al.^17^ investigated the relation of temperature and thermal conductivity of materials and found that the temperature is higher for higher values of thermal conductivity parameter ε. Troyanchuk et al.^18^ found that pure ferromagnetic states develops in certain ceramics in the range 0.2 < x < 0.4. Utilizing the first and second laws of thermodynamics. They found a decrease in thermodynamic parameter with increasing the magnetic parameter.

This paper reports the synthesis of manganese ferrite via solid state reaction route. Kundu et al.^19^ synthesized manganese ferrite nano particles through solid state reaction route using Ti^+4^ ion as dopant and found that the particle diameter decreases with increasing the content of dopant (Ti^+4^). The Solid-state reaction route is less time consuming compared to the other synthesis techniques. Moreover, samples can be heated without melting and ores such as sulphide ore (if present) can be transformed into oxides and will escape as a gas. Solid state reaction route synthesis techniques are usually less expensive and appropriate for enormous productions^20^. Solid state reaction route can achieve the reduction of a compound, which cannot be occur in the presence of water i.e. it has got the capacity to extricate the reactive metals which can’t be reduced from watery solutions like Alkaline earth metals, zirconium, titanium etc. The particle size of manganese ferrite formed via this method, will always be in nanometer which have tolerance to high frequencies^21^.

The destinations of this paper are to examine the phase, microstructural, dielectric properties, AC conductivity and tolerance factor of Mn-ferrites synthesized from low grade Mn-ore via solid state reaction route.

## Experimental procedure

Manganese ferrite (MnFe_2_O_4_) has been synthesized via solid state reaction route. The chemical reagents used in the present work were low grade manganese ore and chemical grade ferric oxide (Fe_2_O_3_). For a proper stoichiometric ratio of Mn and Fe as 1:2, the ferric oxide powder (48.84 g) and low-grade Mn ore (24 wt% Mn) were intimately mixed in a pestle and mortar system. The obtained powders were then pressed into a 9 mm pellets at a pressure of 1.5 Mpa, and sintered at different temperatures (1000, 1100 and 1200 °C) for 3 h in a furnace (Nabertherm, LHT04/18, Germany). After sintering, the samples were cooled to room temperature. Phase analysis of the samples was carried out using a PANalytical X-ray diffractometer (XRD) with Cu Kα radiation (λ = 1.5418 Å). X-ray analysis confirmed that single phase Mn-ferrite with high peak intensity has been reported for the samples sintered at 1200 °C while Mn-ferrite phase with less peak intensities along with other phases has been reported for the samples sintered at 1000 and 1100 °C. Therefore, the samples sintered at 1200 °C were selected for microstructural analysis, elemental analysis, and other properties like dielectric properties. The microstructures analysis of the sample was studied using Scanning electron microscope **(**JSM5910 JEOL, Japan) and elemental analysis was carried out through EDX. The Dielectric properties such as dielectric constant and dielectric losses were investigated as a function of temperature and frequency in the temperature range (25–600 °C) and frequency (1, 10, 100, 250 kHz and 1 MHz) using LCR meter KEYSIGHT, E4980A.

## Results and discussion

### Phase analysis

**Figure 1:**
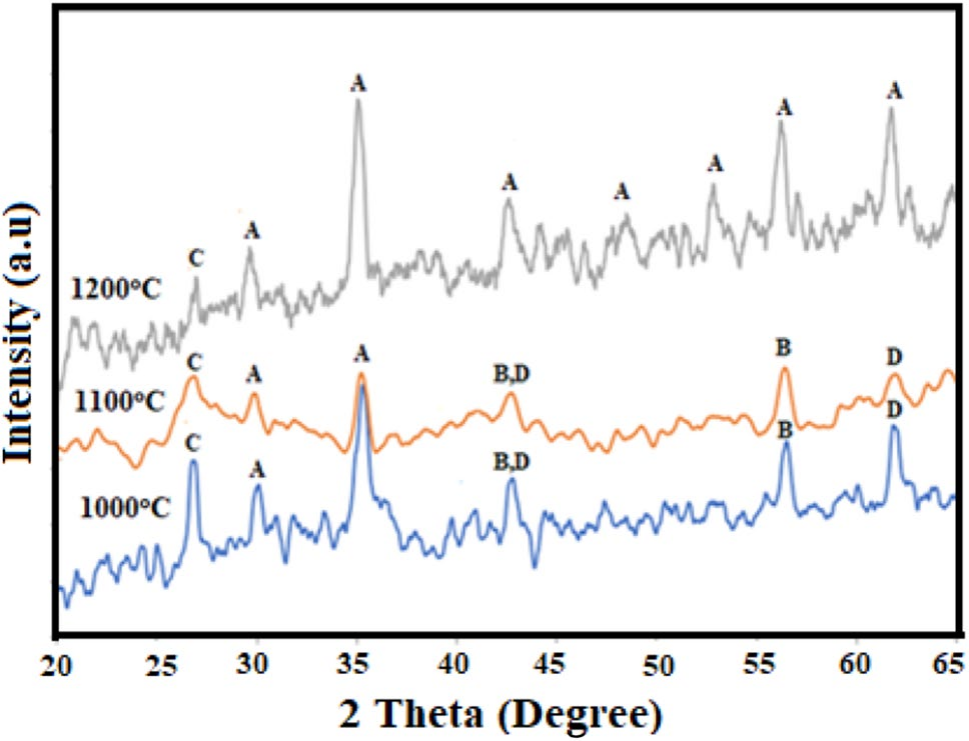
XRD pattern of samples at different sintering temperatures (1000, 1100 and 1200 °C) representing different phases i.e. (A) manganese ferrite (MnFe_2_O_4_), (B) manganese oxide (MnO_2_), (C) silicon oxide (SiO_2_) and (D) iron oxide (FeO).

XRD pattern of the Mn-ferrite synthesized from low grade Mn-ore at different sintering temperatures (1000, 1100 and 1200 °C) is shown in Fig. 1. Various phases such as manganese oxide, silicon oxide and iron oxide have been identified along with manganese ferrite phase for the samples sintered at 1000 and 1100 °C. The peaks labeled as A, B, C and D shows the phase formation of manganese ferrite MnFe_2_O_4_ (JCPDS card no. 74–2403), manganese oxide MnO_2_ (JCPDS card no. 12–141), silicon oxide SiO_2_ (JCPDS card no. 48–476) and iron oxide FeO (JCPDS card no. 46-1312) respectively. Average particle size of the samples prepared at 1000, 1100 and 1200 °C was 5.95 nm, 6.37 nm and 8.275 nm respectively, determined by utilizing Scherrer’s formula (Eq. (1)).

1 $$\mathrm{D }= \frac{0.89\lambda }{\beta \cos\theta },$$

where $\lambda$ is the wavelength of X-rays, $\beta$ is the full width half maxima (FWHM) and $\theta$ is the corresponding diffraction angle.

XRD pattern show that the average particle size of the samples is different for different sintering temperatures and peak intensity also changes with change in temperature as reported in Table 1. The particle size is increasing with rising the sintering temperature which is responsible for high conductivity or low resistivity^22–24^. For the sample sintered at 1200 °C, single phase manganese ferrite is obtained having more prominent phase compared to other samples.

**Table 1:** The measured values of the particle size, lattice constant, density, tolerance factor and bond stretching of the samples sintered at 1200 °C.

Temperature (°C)	Mn:Fe	Time (h)	Particle size (nm)	Lattice constant (A^o^)	Measured density (g/cm^3^)	Tolerance factor	Bond stretching force constant K (Nm^−1^)
1000	1:2	3	5.95	8.534	2.68	1.138	0.237
1100	1:2	3	6.37	8.209	2.70	1.138	0.237
1200	1:2	3	8.24	8.275	2.72	1.138	0.237

The peaks labelled as “C” corresponds to SiO_2_ (JCPDS card no. 48-476) and no other peaks corresponding to impurities can be found, indicating the composition of SiO_2_ in the samples which confirm the association of SiO_2_ with manganese ferrite nanoparticles^25^.

Similarly, using Eq. (2), the bulk density was measured to be 2.72 g/cm^3^. Bulk density demonstrates that how much weight of the material can be pressed per unit area. Material with high bulk densities are difficult to be transported. The reported bulk density of the sample is very low which indicates that large amount of the sample can be packed in a unit area hence its transportation will be easy. Table 1 show an expansion in bulk density with rise in sintering temperature from 1000 to 1200 °C which is due to increase in thermal conductivity^26^.

2 $$\rho_{{\text{m}}} = \frac{m}{{{\uppi }r^{2} {\text{h}}}},$$

where m, r and h represents the mass, radius and thickness of the pellet respectively.

The lattice constant decreases with increasing the sintering temperature as evident from Table 1, it is because of the presence of imperfections and impurities. These imperfections are generated at the time of preparation of sample and change according to the synthesis methods and reagents^27^.

The bond stretching force constant (K), tolerance factor (T) and lattice constant (A) of the spinel structure were derived from Eqs. (3)–(5) respectively and are given in Table 1^28^ and remains the same. The bond stretching force constant will help in building vibrational eigen frequencies and eigen vectors of solids obtained within local density approximation to density functional theory^29^ and the reported tolerance factor shows that the samples have tolerance to high temperatures like 1200 °C. The reported particle size is in acceptable concurrence with recently reported experimental results. The reported tolerance factor remains the same for different sintering temperatures as reported in Table 1 which show that the material show tolerance to high temperatures.

3 $${\text{K}}_{{{\text{ab}}}} = {\text{x}}_{{\text{a}}} {\text{x}}_{{\text{b}}} /{\text{r}}_{{\text{e}}}^{{2}} ,$$

where r_e_ = (r_a_ + r_o_)^2^ + (r_b_ + r_o_)^2^ + 1.155(r_a_ + r_o)_ and x_a_ and x_b_ are the electronegativities of a and b sites^6^.

4 $$\mathrm{T}= \frac{1}{\sqrt{3}}\frac{{r}_{a}+{r}_{o}}{{r}_{b}+{r}_{o}}+\frac{1}{\sqrt{2}}\frac{{r}_{o}}{{r}_{b}+{r}_{o}},$$

5 $$\mathrm{L}= \frac{2\sqrt{3}}{3}({r}_{a}+{r}_{o)}+2({r}_{b}+{r}_{o)}+\sqrt{2}{r}_{o}.$$

The ionic radii of the corresponding Fe, Mn and O ions were taken from the Shannon ionic radii table^30^.

### Microstructural and chemical analyses

**Figure 2:**
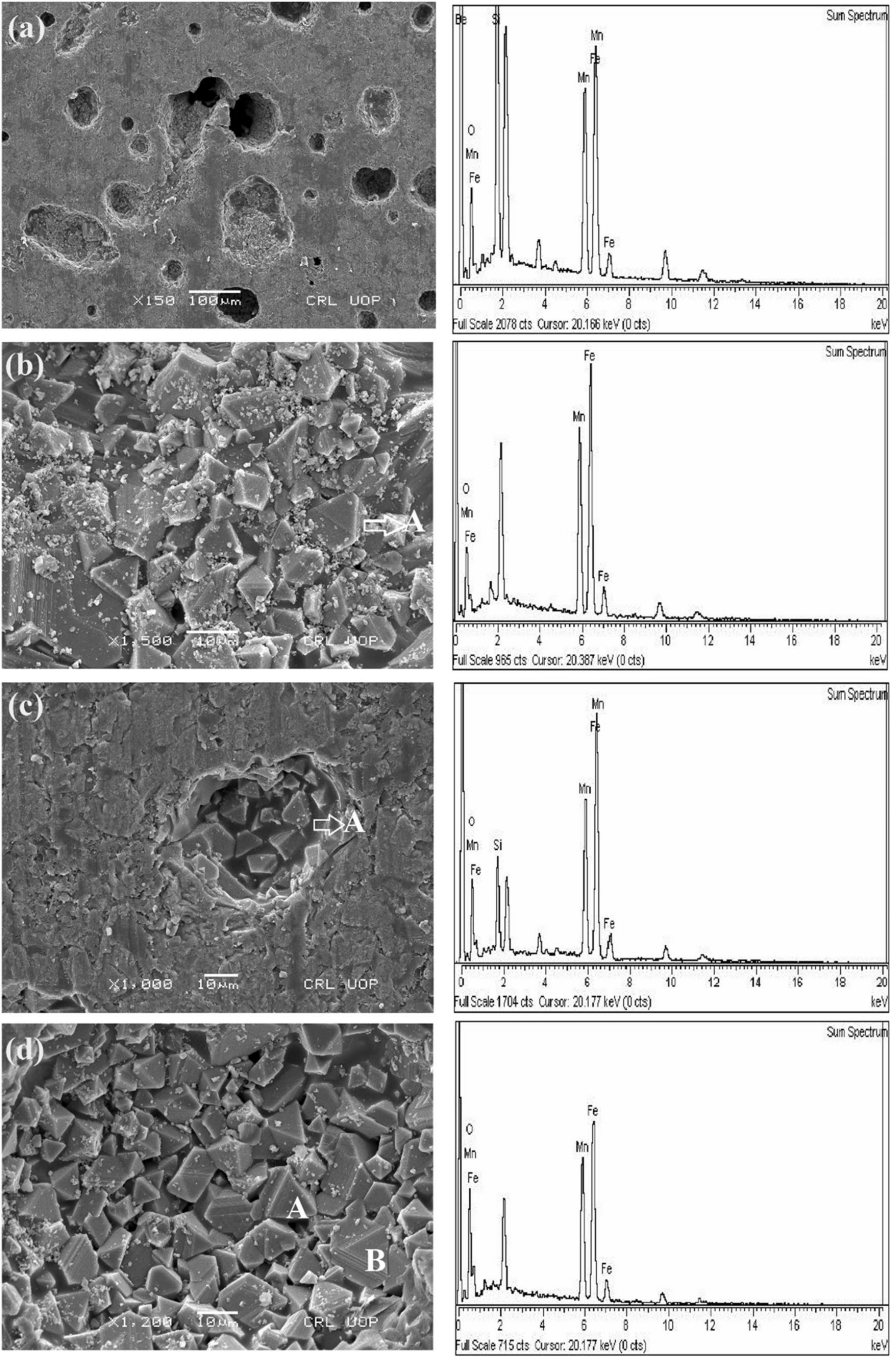
SEM micrographs of the manganese ferrite showing a spongy type, fragile and fractured surface (**a**), highly compact pyramidal and triangular grains (**b–d**).

Figure 2a–d shows the secondary electron images (SEIs) of the manganese ferrite sample sintered at 1200 °C.

It can be seen in Fig. 2a, that at low magnification the overall surface of the sample was found to have large number of pores and voids uniformly distributed. It could be credited to the removal of huge amount of gases during the combustion process. Microstructure images of the non-sintered samples (room temperature) are attached in Supplementary Information which show a smooth and non-porous surface with no grains and consists of Fe_2_O_3_, SiO_2_ and Mn particles along with other impurities due to Mn-ore. This confirms that Mn-ferrite nanoparticles are not synthesized at room temperature. Figure 2b,d shows the highly compact pyramidal and rectangular grains of varying sizes (4–20 µm), and an average grain size of 5.40 µm exhibiting approximately fine-grained microstructure. Ahmed et al.^31^ found that SEM analysis of manganese ferrite nanoparticles prepared through ceramic technique show porosity which is due to the fact that incomplete solid–solid reaction has occurred. Figure 2b showed microstructures with very small compact grains having closed porosity which is indicating a complete solid–solid reaction between varying constituents. The constituents, as evident from the corresponding EDS spectra in Fig. 2b, are Mn, Fe and Yin et al. ^32^ found that the grains are stacked with thick laminar structured silica. This behaviour is evident from Fig. 2c where the grains are stacked in a plain uniform surface of silica as confirmed from the corresponding EDX spectra.

The EDX analysis shows the exact composition of the sintered manganese ferrite determined from various areas is shown in Table 2. The content of silica determined by EDX may be due to manganese ore. The pyramidal and triangular shape grains labeled as ‘A’ and ‘B’ in Fig. 2 contain the high concentration of Mn and Fe along with oxygen. Table 2 show the elemental composition by weight percent of whole surface of Fig. 2b–d and also for the selected regions of Fig. 2b–d labelled as “A” and “B” respectively and confirm the oxygen excess and consequently the excess of oxygen corresponds to decrease in curie point or curie temperature, increase in magnetic moment and high resistivity^33–35^.

**Table 2:** The elemental composition of the manganese ferrite grains as shown in Fig. 2.

Micro region		Elements (wt%)			
		O	Mn	Fe	Si
(b)	Total region	30.14	25.44	34.73	9.69
	A	26.22	30.38	43.40	–
(c)	Total region	26.84	23.78	29.94	19.44
	A	29.47	23.21	36.53	10.80
	Total region	32.27	26.59	37.24	3.91
(d)	B	41.86	23.75	34.39	–

### Dielectric properties

**Figure 3:**
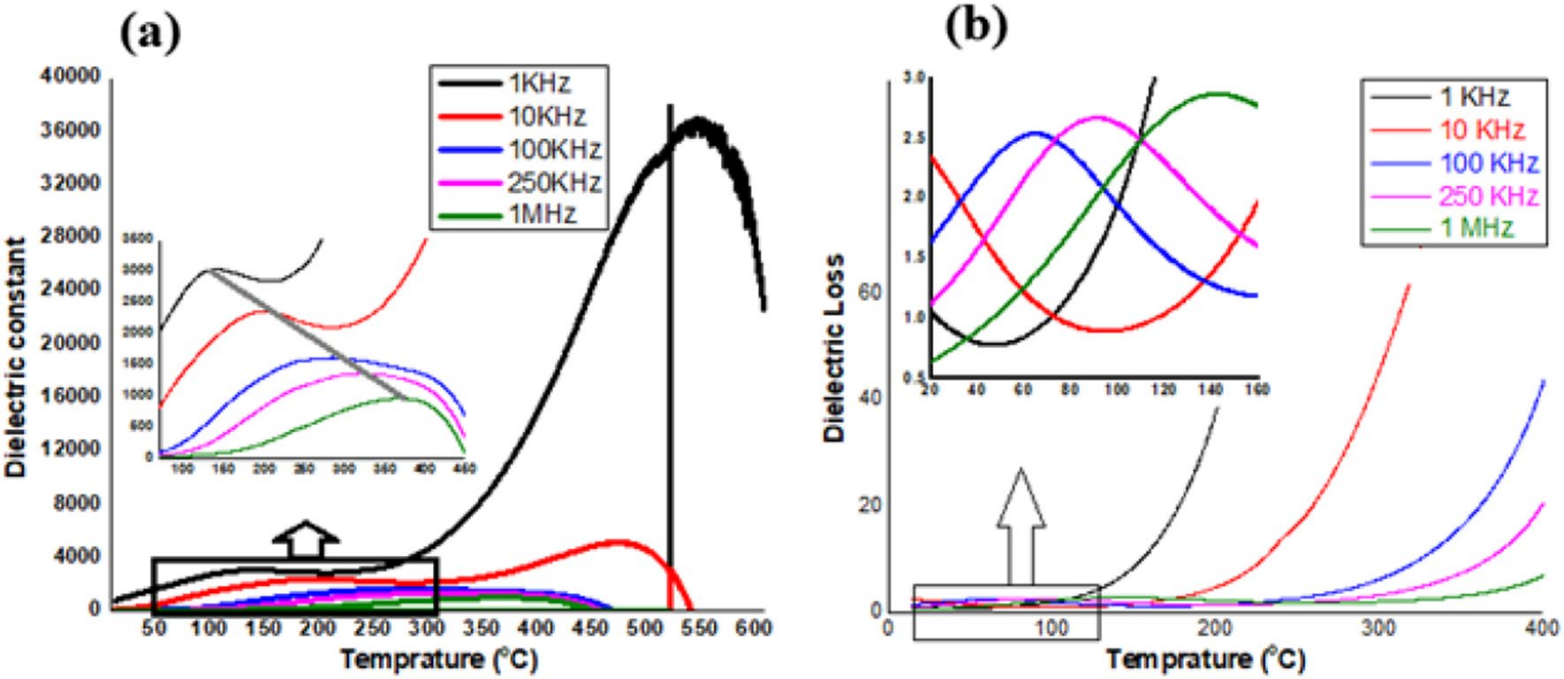
(**a**) Variation of dielectric constant (**a**) and dielectric loss (**b**), at various frequencies and varying temperatures (25–600 °C). (**b**) zoomed region of (**a**) with temperature range (25 to 400 °C).

Figure 3a,b shows the dielectric properties of the manganese ferrite sample, determined in a temperature range (25–600 °C) at various frequencies (1, 10, 100, 250 kHz and 1 MHz). Figure 3a clearly shows an inhomogeneous dielectric response that is greatly depending upon the applied frequencies. The broad peaks also determine the relaxer behavior of the material. Furthermore, it also clearly shows a high dielectric constant (36,000) at low frequency (1 kHz) with a transition temperature of ~ 140 °C. Batoo et al.^3^ observed that ε initially rise gradually while εʹʹ showed small change at low temperature while (εʹ) abruptly rise at high temperature by using solution combustion technique. Further, the increase in (εʹ) with the rise in temperature is very large at small frequencies and hence small for larger frequency. From Fig. 3a we observed that dielectric constant is high for 1 kHz and smaller for 10 kHz frequencies. It is also evident that at same temperature the curves having lower frequencies corresponds to high dielectric constant i.e. the (εʹ) at 1 kHz frequency is higher than at 10 kHz frequency for same temperature. An exponential decrease in dielectric constant have been observed at higher applied frequencies (10, 100, 250 kHz and 1 MHz), which might be due to the space charge polarization^36^.

The modification in space charge polarization happens because of the presence of higher conductivity phases (grains) in the insulating matrix (grain limits) of a dielectric material that introduces restrict accumulation of charge affected by an electric field^37^. Also, it has been seen that a dielectric takes some period to arrange their axes corresponding to an electric field in the space charge polarization. Finally, a point arrive at where the space charge carrier can't support the field and in this way the variation of the direction lingers behind the field, when the frequency of field inversion is raised, and thus εʹ deceases^3^. Figure 3b shows the dielectric loss at different frequencies and varying temperatures (25–400 °C). It clearly shows the frequency dependency of dielectric losses that rises with rising the applied frequency.

### Tangent loss

**Figure 4:**
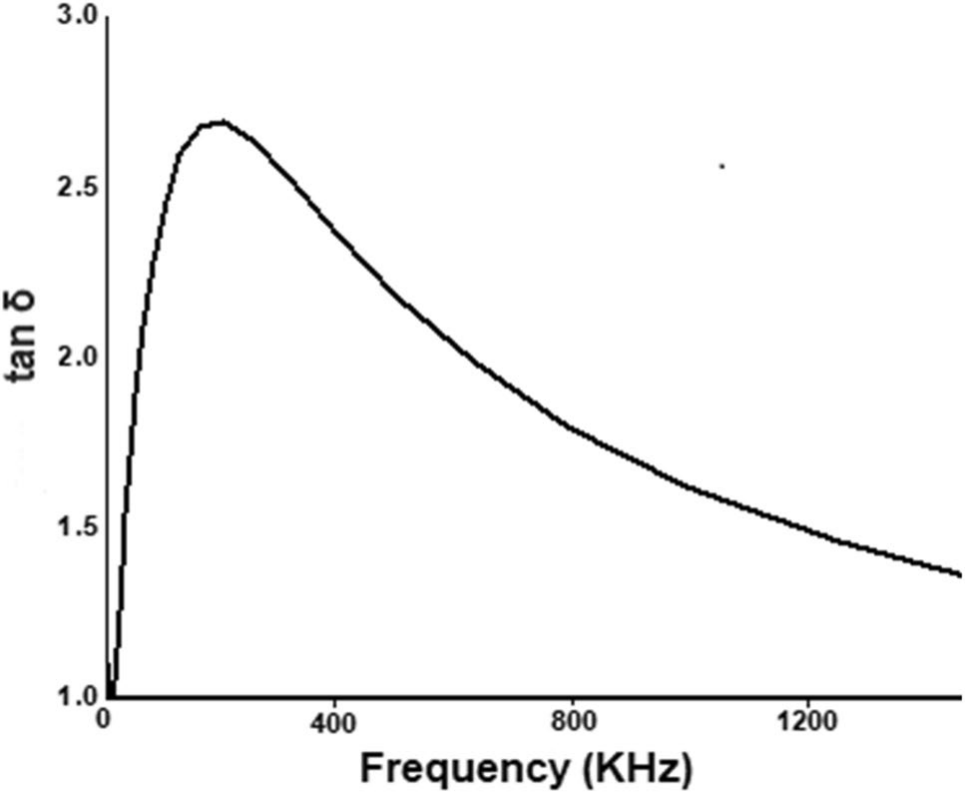
The change of tangent δ with change in frequency at room temperature (25 °C).

The variation of the tangent δ as a frequency function at normal temperature (25 °C) is depicted in Fig. 4, which shows the energy loss in a dielectric^3^. The height of the peak shift to lower value as the frequency gradually increases. Abdeen et al., observed that an ordinary dielectric behavior in ferrites decreases sharply at smaller frequencies and a bit gradually at larger frequencies^38^. Ali et al., reported that the decrease in tangent δ happens as the hooping frequency of electric charge carrier can’t follow the change of applied electric field after a specific critical frequency^39^. The peak behavior can be handily concentrated through Rezlescuu model^40^, as indicated by which the peaking behavior will happen when the charge hooping frequency between two valence state of a similar component related with the applied field frequency  where  is the relaxation time for hooping procedure and ω is the angular frequency.  is inversely proportional to the hooping probability per unit time (P), as depicted by the relation ^3^. Figure 4 shows a very sharp increase in tangent δ at lower frequency then relax for a while making a broad peak and gradually shifted towards the lower values.

### Alternating current (AC) conductivity

**Figure 5:**
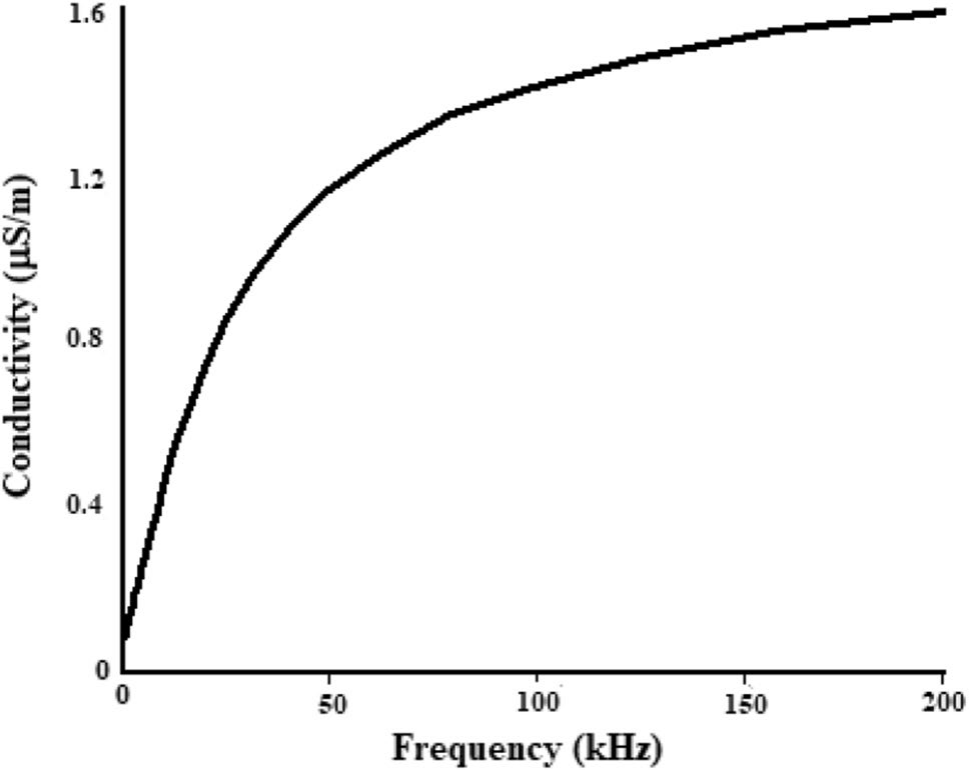
The change in AC conductivity with frequency at normal temperature (25°).

AC (alternating current) conductivity of the sample as frequency function at room temperature is depicted in Fig. 5. Initially the AC conductivity has been reported to increase linearly in accordance to the power law equation (σ_AC_ = Aω^n^), however, with further increase in frequency it gradually increases due to ordinary behavior of ferrites and in the end came to a constant value^3^. Batoo et al.^3^ reported that the rise in AC conductivity with the applied field might be because of the pumping force of applied field that advances the motion of charge transporter among the two Fe ion states and the separation of charge from various trapping centers. In this manner the charge bearers takes an interest in the conduction procedure alongside electrons that are produced from valence interchange of various metal particles^3^. Shirsath et al., reported that the linear change of conductivity with frequency account for the polaron type conduction which is because of ionic movement that depends upon the angular frequency^41^. The frequency independent behavior can be seen at above 180 kHz. The current study shows a sharp increase in ac conductivity at low frequency then reaches to a uniform slope from 30 to 70 Hz and shows least variation at high frequencies as evident from Fig. 5.

### Change of dielectric constant (εʹ) and losses (εʹʹ) with frequencies

**Figure 6:**
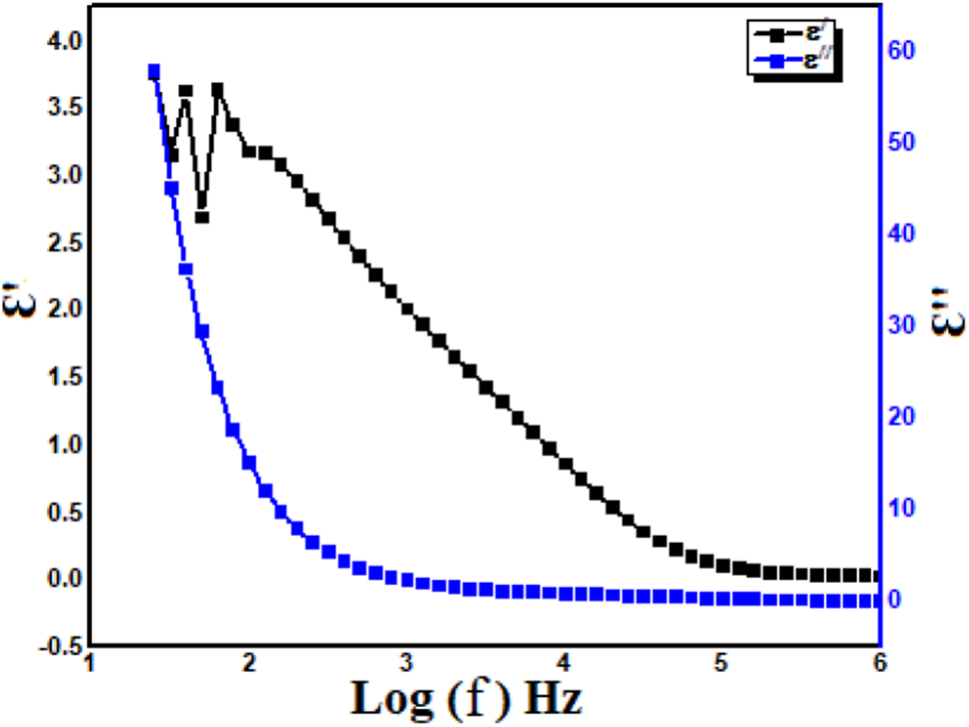
Variation of dielectric constant (εʹ) and losses (εʹʹ) with frequency.

The change in (εʹ) and (εʹʹ) as frequency function for the sample at room temperature is displayed in Fig. 6. Batoo et al.^3^ observed the strong relation between permitivity and frequencies. A strong frequency reliance is depicted for the samples; therefore, the change in dielectric properties of the present framework might be because of the change in frequency. It is additionally evident that both (εʹ) and (εʹʹ) falls with rise in frequency. The large value of dielectric parameters at lower frequencies might be because of Maxwell–Wagner interfacial type of polarization^42,43^ for the non-uniform double layered dielectric framework which agrees with Koop’s theory^44^. (εʹ) and (εʹʹ) falls with rise in frequency and attain a constant value because of the fact that after a specific frequency of the exterior electric field, the electron interchange between Fe^2+^ and Fe^3+^ can’t follow the alternating field. The non-uniform dielectric framework is composed of two layers. One layer is the genuinely best conducing large ferrite grains which are isolated by another layer having ineffectively conducting grain boundaries. The grain boundaries with smaller and larger conductivities are observed to be successful at smaller frequencies while the ferrite grains having large conductivity and lower (εʹ) are progressively efficacious at higher frequency^45,46^.

## Conclusion

The present work reports the successful Synthesis of single-phase spinel Mn-ferrite from low grade Mn-ore via solid state reaction route. The synthesis took place at a sintering temperature of 1200 °C. The average particle size of the synthesized manganese ferrite nanoparticles is in acceptable concurrence with recently reported experimental results. It has been observed that particles size of the synthesized nanoparticles increases with increasing the sintering temperature (Table 1) which accounts for best conductivity of the material at high temperatures while manganese ferrite phase becomes more relevant at high temperature i.e. 1200 °C. Microstructural SEM images confirmed that the obtained manganese ferrite nanoparticles have porous and spongy type surface morphology with some regions having pyramidal and triangular shaped grains of average crystallite size 5.40 μm. The porosity appears due the exchange of high amount of gasses during heat treatment of the samples. Moreover, varying concentrations of iron and manganese have been found in the synthesized manganese ferrite nanoparticles along with some content of silica as an impurity. The nanoparticles in certain regions are found to be imbedded in the thick layers formed by silica. The sample possess high dielectric constant (εʹ) and less dielectric loss (εʹʹ) at low frequencies and low temperature which accounts for best conducting properties of the synthesized manganese ferrite. This material is found to be reliable with high frequencies because its tangent losses were found to decrease with increasing frequencies. Finally, the obtained Mn-ferrite nanoparticles behaves like normal ferrites i.e. their AC conductivity increase linearly at start and gradually at end with increase in frequency which accounts for the reliability of synthesis of manganese ferrite nanoparticles through solid state reaction route.

## Supplementary Information

Supplementary Information 1.

Supplementary Information 2.
