# Supplementary material for: Synthesis and characterization of manganese ferrite from low grade manganese ore through solid state reaction route
Source: Sci Rep. 2021 Aug 10;11:16190. doi: 10.1038/s41598-021-95625-z (PMC8355231; doi:10.1038/s41598-021-95625-z)
Supplement: Supplementary file 1 — Supplementary Information 1. [file 41598_2021_95625_MOESM1_ESM.pdf]

Title: "Synthesis and characterization of Manganese Ferrite from low grade Manganese Ore through solid state reaction route".

Authors detail:

1- Salar ahmad

Affiliation: Materials research laboratory, department of physics, university of Peshawar, 25120, KPK, Pakistan.

2- Sajjad ali

Affiliation: Materials research laboratory, department of physics, university of Peshawar, 25120, KPK, Pakistan.

3- Ikram Ullah

Affiliation: Department of Sciences and Humanities, National university of computer and emerging sciences, (KPK), 25000, Pakistan

4. M.S. Zobaer

Affiliation: McGovern Medical School, The University of Texas Health Science Center at Houston, Texas, USA

5. Ashwag Albakri

Affiliation: Department of Computer Science, College of Computer Science & Information Technology, Jazan University, Jazan 45142, Saudi Arabia

6. Taseer Muhammad

Affiliation: Department of Mathematics, College of Sciences, King Khalid University, Abha 61413, Saudi Arabia

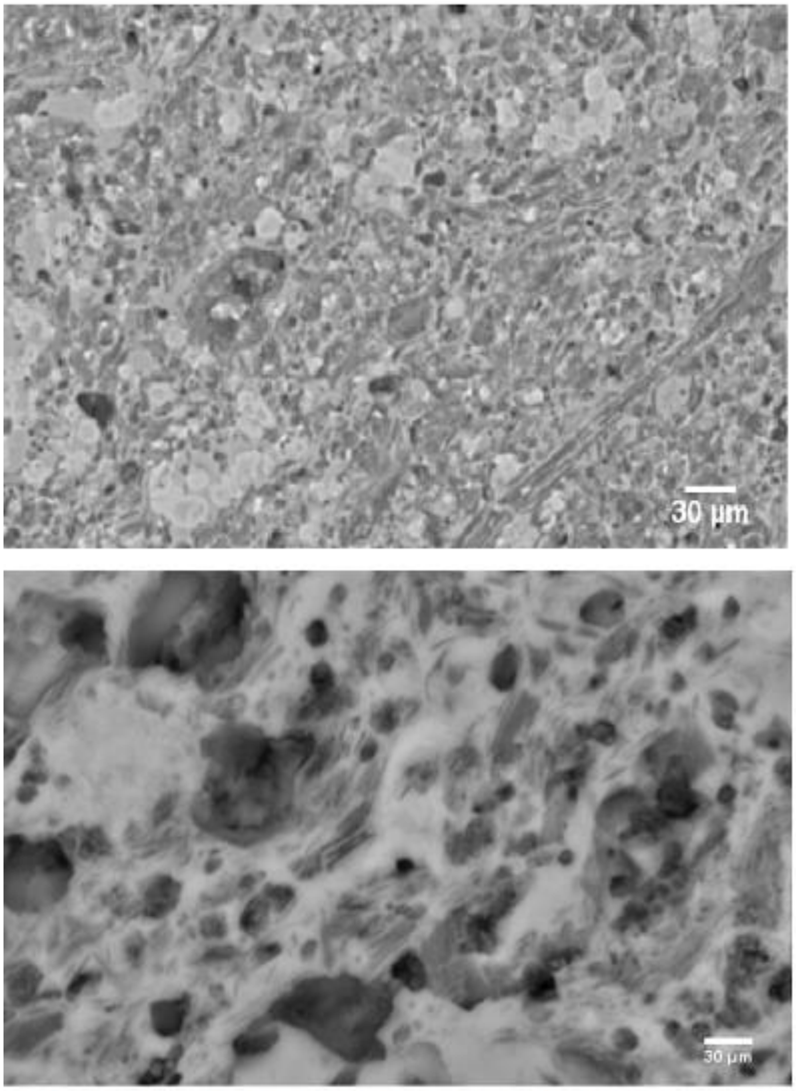

Figure(1,2): SEM image of samples at room temperature
