# Supplementary material for: Synthesis and characterization of manganese ferrite from low grade manganese ore through solid state reaction route
Source: Sci Rep. 2021 Aug 10;11:16190. doi: 10.1038/s41598-021-95625-z (PMC8355231; doi:10.1038/s41598-021-95625-z)
Supplement: Supplementary file 2 — Supplementary Information 2. [file 41598_2021_95625_MOESM2_ESM.pdf]

Title: "Synthesis and characterization of Manganese Ferrite from low grade Manganese Ore through solid state reaction route".

Authors detail:

1- Salar ahmad

Affiliation: Materials research laboratory, department of physics, university of Peshawar, 25120, KPK, Pakistan.

2- Sajjad ali

Affiliation: Materials research laboratory, department of physics, university of Peshawar, 25120, KPK, Pakistan.

3- Ikram Ullah

Affiliation: Department of Sciences and Humanities, National university of computer and emerging sciences, (KPK), 25000, Pakistan

4. M.S. Zobaer

Affiliation: McGovern Medical School, The University of Texas Health Science Center at Houston, Texas, USA

5. Ashwag Albakri

Affiliation: Department of Computer Science, College of Computer Science & Information Technology, Jazan University, Jazan 45142, Saudi Arabia

6. Taseer Muhammad

Affiliation: Department of Mathematics, College of Sciences, King Khalid University, Abha 61413, Saudi Arabia

Peak peakposition(2theta)	Full width half maxima(FWHM)	Crystallite size D (nm)	average D(nm)
26.85677	0.40783	20.02693031	5.956356874
28.77308	32.38303	0.25326458	
35.33846	0.82323	10.12793431	
42.7954	0.51088	16.70142721	
45.49877	17.41785	0.49457591	
56.61585	15.04268	0.599851816	
62.43211	8.55303	1.086069617	
62.43459	26.87264	0.345679019	
56.61585	316.48944	0.028510837	
61.87422	0.58521	15.82674585	
61.87422	320.08301	0.02893615	

Table 1: Particle size of samples sintered at 1000 ^{0}C

peak position (2 Theta)	Full width half maxima(FWHM)	Crystallite size D(nm)	average D(nm)
27.56437	3.37738	2.421936086	6.37129303
35.24111	52.18313	0.159732976	
35.24111	0.49582	16.81127558	
42.12577	3.35587	2.536772037	
56.38127	0.57435	15.69332194	
64.76223	15.55604	0.604719566	

Table 2: Particle size of samples sintered at 1100 ^{0}C

Peak position (2 theta)	Full width half maxima(FWHM)	Crystallite size D (nm)	Average D (nm)
29.96772	0.88877	9.253139472	8.24172507
35.274	0.95221	8.754504713	
56.51728	0.6992	12.89931854	
62.05792	0.84003	11.03637925	
47.44147	474.09654	0.01830298	
47.75187	1.18745	7.316309495	
22.32652	0.96237	8.414121066	

Table 3: Particle size of samples sintered at 1200 ^{0}C
